# Taste Evaluation of Yellowtail (*Seriola Quinqueradiata*) Ordinary and Dark Muscle by Metabolic Profiling

**DOI:** 10.3390/molecules24142574

**Published:** 2019-07-15

**Authors:** Ryota Mabuchi, Ayaka Ishimaru, Miwako Adachi, Huiqing Zhao, Haruka Kikutani, Shota Tanimoto

**Affiliations:** 1 Faculty of Human Culture and Science, Prefectural University of Hiroshima, 1-1-71, Ujina-Higashi, Minami-ku, Hiroshima 734-8558, Japan; 2 Graduate School of Comprehensive Scientific Research, Prefectural University of Hiroshima, 1-1-71, Ujina-Higashi, Minami-ku, Hiroshima 734-8558, Japan

**Keywords:** GC-MS, electronic tongue, metabolomics, metabolic profile, taste attribute, dark muscle, ordinary muscle, fish meat, PCA, O2PLS

## Abstract

We performed metabolic profiling on yellowtail (*Seriola quinqueradiata*) muscle to develop an objective taste evaluation method for fish meat. Dark (DM) and ordinary (OM) muscle samples before and after storage were subjected to gas chromatography-mass spectrometry (GC-MS) analysis and taste measurements using an electronic tongue. The metabolites identified by the GC-MS analysis were treated as *x* variables, and the taste values obtained by the electronic tongue were treated as *y* variables. The relationships between the metabolites and taste attributes were evaluated by two-way orthogonal projections to latent structures (O2PLS) analysis. The O2PLS analyses were normalized in two ways, unit variance (UV) and pareto (Par) scaling. The O2PLS (UV) analysis produced 3+1+0 models in Autofit and this model was statistically significant with *R^2^Y* (0.73) and *Q^2^* (0.52) metrics. In particular, significant correlations were found between DM or OM and metabolite intensity and taste attributes, and strong associations were found between “sourness” and lysine, “irritant” and alanine and phenylalanine, “saltiness” and pantothenic acid, and “umami” and creatinine and histidine. The O2PLS (Par) analysis of DM generated significant predictive models for “acidic bitterness,” “irritant,” “saltiness,” “bitterness,” “astringency,” and “richness.” Among these, only “irritant” was affected by storage. This method was thus effective in evaluating the taste of yellowtail muscle.

## 1. Introduction

Fish meat is generally recognized as a healthy food that is rich in high-quality proteins and *n*-3 fatty acids such as eicosapentaenoic acid and docosahexaenoic acid, and the demand for marine products is increasing worldwide. However, this food type is significantly more susceptible to rotting and degeneration than mammalian meat, and thus, aging, which is generally performed for livestock meat, is not performed for fish meat. However, in Japan, where people eat raw fish (*sashimi*), the taste is thought to improve if the meat is stored for a short time rather than being immediately consumed after catching. It is thus important to objectively evaluate the taste of such empirically assessed food using chemical methods. To evaluate the taste of fish meat, analyses of taste components and sensory characteristics have been conducted. However, while it is also necessary to analyze the relationship between taste components and sensory evaluation by humans to objectively assess such taste attributes [1], sensory evaluation is labor-intensive and requires training, and thus it is difficult to obtain reproducible data.

Recently, biosensors have been developed that can chemically measure taste. For example, the electronic tongue quantitatively measures taste based on the electrical potential responses of artificial lipid membranes and takes into account human taste threshold values [2]. This can be applied to various foods [3] and is considered very effective especially for foods that are difficult to assess by sensory evaluation. In addition, for the analysis of taste components and other attributes, metabolomics has been developed to analyze more factors comprehensively [4]. Methods such as NMR, GC-MS, and LC-MS, among others, have been developed for such metabolomics approaches, and in particular, the analysis of water-soluble primary metabolites by GC-MS is widely applied [5]. Further, the electronic tongue and metabolomics can be combined to provide an objective taste evaluation, and to date have been applied to Japanese sake [6], coffee [7], and whitefish [8], among other foods. Previously, we analyzed the relationships between the water-soluble primary metabolic components of four whitefish species and the taste attributes obtained by the electronic tongue [8] and identified differences in the metabolic components among different types of yellowtail muscle [9]. However, it is still unclear how the metabolic components of these muscle types affect the taste attributes of fish meat, and to the best of our knowledge, no studies have investigated the effects of storage on taste attributes using an electronic tongue and metabolic profiling.

Therefore, in this study, we applied a new taste evaluation method to yellowtail dark muscle (DM) and ordinary muscle (OM) before and after storage. The relationships between the metabolites and taste attributes were evaluated by two-way orthogonal projections to latent structures (O2PLS) analysis, in which *x* to *y* and *y* to *x* can be predicted with *x* as the metabolic component and *y* as the taste value. Significant correlations were found between DM or OM and the intensity of metabolites and taste attributes, whereas OM storage had no effect on taste and there were no significant relationships with metabolites. However, in DM, storage affected the taste attribute “irritant,” which was related to a metabolic component. Therefore, this method was very effective in evaluating the taste of fish meat.

## 2. Results

### 2.1. Electronic Tongue

**Figure 1:**
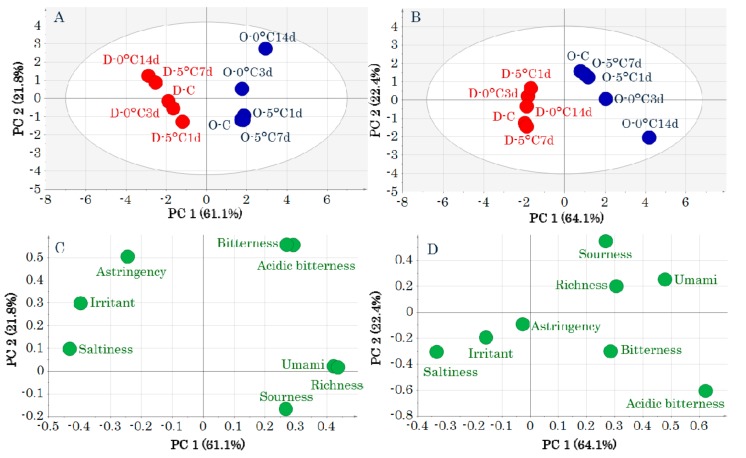
Score plots (**A**,**B**) and loading plots (**C**,**D**) obtained by a principal components analysis of taste-attribute profiles. Data were pretreated with unit variance scaling (**A**,**C**) and pareto scaling (**B**,**D**). Symbols in score plots (**A**,**B**) indicate sample IDs (Table S1).

The values of each taste attribute obtained by the electronic tongue are shown in Table S1. The results of a principal components analysis (PCA)-Y are shown in Figure 1. The unit variance scaling (UV) and pareto scaling (Par) gave similar results. DM is to the left and OM is to the right of the first principal component in the score plots of Figure 1A,B. “Astringency,” “irritant,” and “saltiness” are to the left and “bitterness,” “acidic bitterness,” “richness,” “sourness,” and “umami” are to the right of the first principal component in the loading plots of Figure 1C,D. “Astringency,” “irritant,” and “saltiness” were related to DM, and “bitterness,” “acidic bitterness,” “richness,” “sourness,” and “umami” were related to OM.

**Figure 2:**
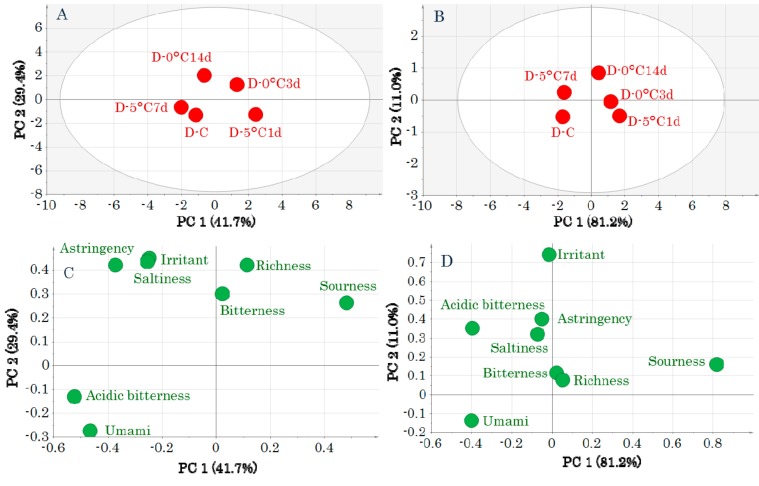
Score plots (**A**,**B**) and loading plots (**C**,**D**) obtained by a principal components analysis of taste-attribute profiles in dark muscle samples. Data were pretreated with unit variance scaling (**A**,**C**) and pareto scaling (**B**,**D**). Symbols in score plots (**A**,**B**) indicate sample IDs (Table S1).

**Figure 3:**
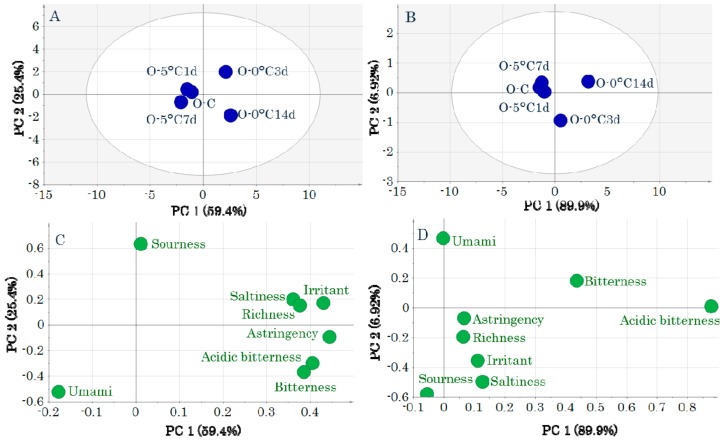
Score plots (**A**,**B**) and loading plots (**C**,**D**) obtained by a principal components analysis of taste-attribute profiles in ordinary muscle samples. Data were pretreated with unit variance scaling (**A**,**C**) and pareto scaling (**B**,**D**). Symbols in score plots (**A**,**B**) indicate sample IDs (Table S1).

Subsequently, PCA-Y was performed for both muscle types to evaluate the effects of storage on the taste attributes (Figure 2 and Figure 3). In the DM score plots, the samples before storage are shown lower left of the center, and there was no significant effect of storage (Figure 2A,B). The loading plots (Figure 2C,D) show that samples before storage were related to “umami.”

In contrast, in the OM score plot, the samples before storage and storage at 5 °C are to the left of the first principal component and 0 °C storage is to the right. The before storage and 5 °C storage groups were indistinguishable because of their overlapping positions (Figure 3A,B). This indicates that the taste attributes after 5 °C and 0 °C storage differed. Most taste attributes were related to 0 °C storage (Figure 3C,D).

### 2.2. Gas Chromatography-Mass Spectrometry (GC-MS) Analysis

We annotated 88 metabolic components by GC-MS analysis (Table S1). The results of PCA-X are shown in Supplementary Figures S1 and S2. The PCA-X, which included all samples, revealed that DM and OM were completely separate (Supplementary Figure S1), consistent with the results of our previous research [9]. The PCA-X for each muscle type separated the samples before and after storage in terms of their metabolic components (Supplementary Figure S2).

### 2.3. Two-Way Orthogonal Projections to Latent Structures (O2PLS)

**Figure 4:**
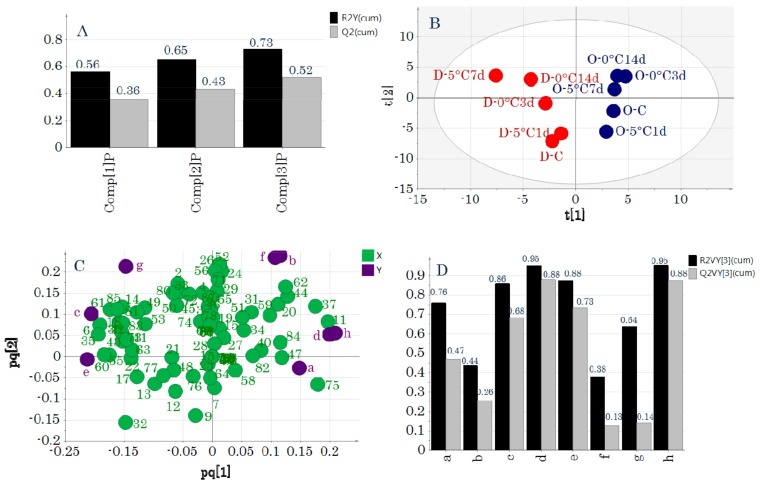
Two-way orthogonal projections to latent structures analysis with unit variance scaling as a pretreatment. (**A**) Evaluation of the model when Autofit was applied. Model dimensionality was 3+1+0. (**B**) Score plot. Symbols indicate sample IDs (Table S1). (**C**) Loading plot. Numbers and letters indicate variable IDs (Table S1). (**D**) Evaluation of the predictive model for each *y* variable. Letters on the *x*-axis indicate *y* variable IDs (Table S1).

O2PLS analysis was performed next to investigate the relationships between the primary metabolic components and taste attributes. Figure 4 shows the model obtained by O2PLS analysis using UV-pretreated data. Applying Autofit resulted in 3+0+1 model dimensions (Figure 4A). Autofit uses cross-validation rules to automatically adjust the model to determine the number of significant components. Because the *R^2^Y* value of this model was 0.73 and the *Q^2^* value was 0.52, it was statistically significant. The score plot is shown in Figure 4B and the loading plot is shown in Figure 4C. The score plots and loading plots obtained were similar to those obtained by PCA-X (Supplementary Figure S1) and PCA-Y (Figure 1). Figure 4D shows the cumulative *R^2^* and *Q^2^* values for individual *y* variables. Predictive models that included significant taste attributes were created for “irritant,” “umami,” “saltiness,” and “richness.”

**Figure 5:**
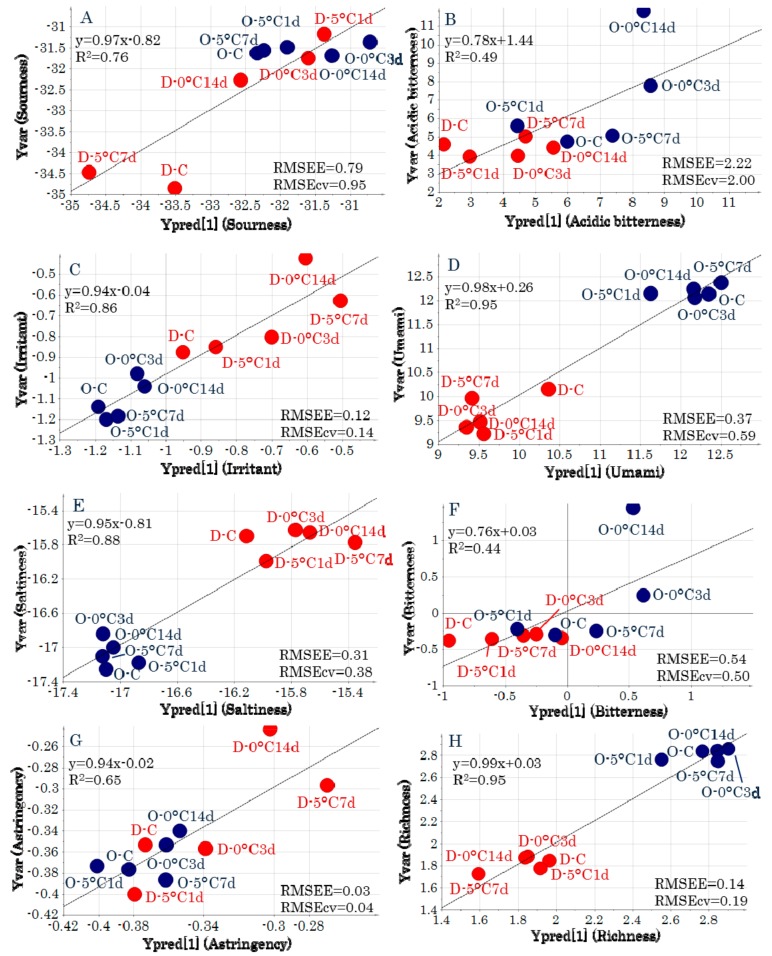
Regression analyses of the predictive model for each *y* variable. (**A**) Sourness. (**B**) Acidic bitterness. (**C**) Irritant. (**D**) Umami. (**E**) Saltiness. (**F**) Bitterness. (**G**) Astringency. (**H**) Richness. Symbols indicate sample IDs (Table S1). *RMSEE*, root mean square errors of estimation; *RMSEcv*, root mean square errors of cross-validation.

The regression analyses derived from the predictive models for each taste attribute had high *R^2^* values and low root mean square errors of estimation (*RMSEE*) and root mean square errors of cross-validation (*RMSEcv*) values (Figure 5). The metabolites that were related to taste attributes for which significant models could be generated were identified from the loading plots (Figure 4C). “Sourness” was located near stearic acid and lysine, and therefore, these metabolic components were highly related to “sourness.” In addition, “irritant” was related to alanine, glycine, and phenylalanine, “saltiness” was related to pantothenic acid and *N*-acetylmannosamine, and “umami” was related to creatinine and histidine.

The O2PLS analysis of Par-pretreated data is shown in Supplementary Figure S3. When Autofit was applied to the model, the model had a dimensionality of 1+1+3. In this model, *R^2^Y* was 0.49 and *Q^2^* was 0.29, and a statistically significant model could not be created (Supplementary Figure S3A). In Supplementary Figure S3B, DM is to the left and OM is to the right and are separate, as in the PCA results. However, as shown in Supplementary Figure S3C, galactose and taurine were related to DM and phosphoric acid, and lactic acid were related to OM, and these were important *x* variables for discrimination in the score plot. The taste attributes and many metabolic components were located at the center (near 0), and no relationship between the metabolic components and taste attributes was found. Cumulative *R^2^Y* and *Q^2^* values of the model for each taste attribute are shown in Supplementary Figure S3D. “Umami,” “saltiness,” and “richness” were in the statistically significant model, indicating that the metabolic components can be predicted from the strength of these attributes.

The O2PLS analysis of Par-pretreated data is shown in Supplementary Figure S3. When Autofit was applied to the model, the model had a dimensionality of 1+1+3. In this model, *R^2^Y* was 0.49 and *Q^2^* was 0.29, and a statistically significant model could not be created (Supplementary Figure S3A). In Supplementary Figure S3B, DM is to the left and OM is to the right and are separate, as in the PCA results. However, as shown in Supplementary Figure S3C, galactose and taurine were related to DM and phosphoric acid, and lactic acid were related to OM, and these were important *x* variables for discrimination in the score plot. The taste attributes and many metabolic components were located at the center (near 0), and no relationship between the metabolic components and taste attributes was found. Cumulative *R^2^Y* and *Q^2^* values of the model for each taste attribute are shown in Supplementary Figure S3D. “Umami,” “saltiness,” and “richness” were in the statistically significant model, indicating that the metabolic components can be predicted from the strength of these attributes.

The regression analyses derived from the predictive model for each taste attribute had high *R^2^* values and low *RMSEE* and *RMSEcv* values (Supplementary Figure S4). There were taste attributes that were difficult to predict from the metabolic components after O2PLS analysis using Par as a pretreatment method, and thus, O2PLS analysis was performed again using only predictable taste attributes. As a result, when Autofit was applied, the dimensionality of the model was 2+5+0, *R^2^Y* was 0.97, *Q^2^* was 0.84, and the model had good predictive ability (Supplementary Figure S5).

Next, O2PLS analysis was performed for each muscle type, and the results for the DM samples are shown in Supplementary Figure S6 (UV) and Supplementary Figure S7 (Par). When O2PLS analysis of the DM samples using UV-pretreated data was conducted with Autofit applied, the model had a dimensionality of 1+0+3, *R^2^Y* was 0.25, and *Q^2^* was −0.11, and a statistically significant model could not be created (Supplementary Figure S6). In contrast, when preprocessing using Par, Autofit created a 1+3+3, statistically significant model, because *R^2^Y* was 0.65 and *Q^2^* was 0.55 (Supplementary Figure S7). The cumulative *R^2^Y* and *Q^2^* values of the model for each taste attribute are shown in Supplementary Figure S7D. A significant model was created with many taste attributes.

The regression analyses derived from the predictive model for each taste attribute had high *R^2^* values (Supplementary Figure S8). The “irritant” value increased after storage (Supplementary Figure S8C), and the “richness” value increased after 0 °C and decreased after 5 °C storage (Supplementary Figure S8H). No relationships were found between “acidic bitterness,” “saltiness,” “bitterness,” or “astringency” and storage. The metabolites that were related to taste attributes for which significant models could be generated were identified from the loading plots. “Acidic bitterness” was related to lactitol and maltose, “irritant,” “saltiness,” and “richness” were related to glucose, “bitterness” and “astringency” were related to niacinamide, and “richness” was related to creatinine (Supplementary Figure S7C).

The O2PLS analysis using both UV and Par for OM did not create a significant model, and no significant *R^2^* or *Q^2^* values for any taste attribute were generated (Supplementary Figure S9).

## 3. Discussion

Metabolomics can be applied to various fields, and is important in foodomics [10,11]. For food, this approach is applied for various food quality evaluation methods [4,11]. When evaluating the taste of food using metabolomics, methods based on the correlations between data obtained by sensory evaluation and metabolic components, such as the quantitative descriptive analysis (QDA) method, are used [12]. However, in the case of fish meat, unlike processed products, it is difficult to perform sensory evaluation such as the QDA method. Therefore, we devised a way of using an electronic tongue as an alternative to sensory evaluation. This method has previously revealed taste components related to differences in whitefish species [8]. We believe that the use of metabolomics will result in a unified method of taste evaluation for fish meat. To increase the effectiveness of this method, in this study, we used the yellowtail as a model and evaluated the effects of differences in muscle type and storage on taste attributes. We investigated the relationships between the taste attributes and metabolic components in yellowtail muscle by performing O2PLS analysis, which is a very effective analysis method when used for datasets with many *y* variables, as in this study.

Studies of yellowtail DM and OM components have focused on lipids, *K* values, extractive components, and volatile or flavor components [13,14,15,16,17]. Previously, we found that short-term storage has different effects on these components in DM and OM, which were separated by GC-MS-based metabolomics that targeted primary metabolic components [9]. This was confirmed in the present study by PCA-X (Supplementary Figure S1). The PCA-Y distinguished DM and OM from the values of each taste attribute that were obtained by the electronic tongue (Figure 1). Therefore, an electronic tongue can distinguish between DM and OM, as can other evaluation methods. A previous study utilizing taste tests found that OM “umami,” “richness,” and “astringency” are unaffected by ice storage for 20 days [13]. In contrast, DM is affected by storage because “umami” and “richness” decrease after ice storage [13]. The PCA-Y revealed that OM was unaffected by storage, but DM was affected. This is consistent with the results of the taste test described previously herein. However, PCA-X found that OM was affected by storage. This indicates that components other than metabolic components are involved in the taste changes caused by storage.

O2PLS analysis was performed to evaluate the PCA-X and PCA-Y results, and statistically significant models were obtained under the following analysis conditions—O2PLS (UV) analysis (Figure 4), O2PLS (Par) analysis, in which significant taste attributes were analyzed with *y* variables (Supplementary Figure S5), and O2PLS (Par) analysis of DM (Supplementary Figure S7). The diverse methods of pretreating the same data had differing results on the statistical significance of the model. When using UV, each of the variables (after centering) is divided by its standard deviation, such that the result does not reflect the quantitative contributions of the variables. In contrast, when using Par, the quantitative contributions are considered in the calculations after centering. Therefore, because UV and Par differ in variables that might be important, when performing O2PLS, it is necessary to use different data pretreatment methods.

The O2PLS (UV) analysis created a significant model, and a valid predictive model for the taste attributes “irritant,” “umami,” “saltiness,” and “richness” was generated (Figure 4). Although the O2PLS (Par) analysis did not produce a significant model, effective predictive models for “umami,” “saltiness,” and “richness” were formed (Supplementary Figure S3). “Umami,” “saltiness,” and “richness” were important taste attributes for distinguishing between DM and OM according to both O2PLS (UV) and O2PLS (Par) analyses. Many of the metabolic components that were related to these important taste attributes were amino acids, which are taste components, and many of these are related to the taste of fish and shellfish. Among the components that were found to be related to taste attributes in this study, alanine, glycine, phenylalanine, histidine, and lysine are the main taste components of fish and shellfish [1]. “Umami” and “richness” were related to creatinine and histidine, and creatinine is one of the most important components that determines the taste of yellowtail meat based on omission tests [18]. We also found that creatinine is an important taste component of this type of meat.

The O2PLS (Par) analysis of DM generated significant predictive models for “acidic bitterness,” “irritant,” “saltiness,” “bitterness,” “astringency,” and “richness.” Among these, only “irritant” was affected by storage. Storage did not have a significant effect on taste, and therefore, it is important to consider other factors (e.g., safety aspects) when storing yellowtail. In addition, the storage method used in this study did not increase the number of viable bacteria [19], so is considered safe.

## 4. Materials and Methods

### 4.1. Chemicals

All the reagents used were special-grade chemicals. Methanol, chloroform, pyridine, and ribitol were purchased from Wako (Osaka, Japan). The derivatization reagents methoxyamine hydrochloride and *N*-methyl-*N*-(trimethylsilyl) trifluoroacetamide (MSTFA) were purchased from Sigma-Aldrich (St. Louis, MO, USA) and GL Sciences (Tokyo, Japan), respectively.

### 4.2. Experimental Samples

DM and dorsal OM from a previous study [19] were used. Two yellowtails were purchased at a local market in Hiroshima, Japan on three occasions, July 14, September 29, and November 5, 2014, totaling six fish (mean weight, 5.4 ± 1.2 SD kg). All six fish had been reared by aquaculture and were killed using the *ikejime* fish-slaughtering method. They were then transported on ice to a laboratory within 8 h. Muscle samples of the same type from two fish that were purchased on the same date were minced together using a food processor (MK-K60P, Panasonic, Japan). The minced muscle samples were stored in ice (0 °C) for 14 days or at 5 °C for 7 days, before being stored at −80 °C until analysis. As previously reported, the samples were stored under conditions in which the number of viable bacteria did not significantly increase [19].

### 4.3. Electronic Tongue

#### 4.3.1. Sample Preparation

The method employed is described elsewhere [8]. Briefly, fish meat (5 g) was added to 20 mL ultrapure water and homogenized with an ACE HOMOGENIZER AM-7 (NIHONSEIKI KAISHA Ltd., Tokyo, Japan) at 5,000 rpm for 5 min over ice. After centrifugation (15,000× *g* for 15 min at 4 °C), the supernatant was collected and made up to 70 mL. Half of the sample (35 mL) was used to measure the initial taste and the other half was used to determine the aftertaste (see Section 4.3.2.).

#### 4.3.2. Method of Measurement

Taste was measured using a TS-5000Z taste sensor system (Insent, Japan) following the method described in a previous report [8]. Each sample solution was tested using five types of sensors as follows—AAE, CT0, CA0, C00, and AE1. The differences in human perception of taste intensity were estimated based on Weber’s law from the average of three repeated measurements, and the resultant value was taken as the intensity of each taste attribute. This system detects two types of taste including the initial taste and aftertaste. In this study, the relative potentials obtained from the AAE (“umami”), CT0 (“saltiness”), CA0 (“sourness”), C00 (“acidic bitterness”), and AE1 (“irritant”) sensor probes were used to measure the selective initial tastes. The changes in membrane potential caused by adsorption values obtained from the C00 (“bitterness”), AE1 (“astringency”), and AAE (“richness”) sensor probes were used to measure selective aftertastes [20].

### 4.4. GC-MS Analysis

#### 4.4.1. Pretreatment

Sample preparation was conducted as described in previous studies [8,9]. Briefly, fish fillets were freeze-dried and powdered in a mill. Mixed solutions of methanol/ultrapure water/chloroform (2.5/1/1 v/v/v, 1 mL) and ribitol (internal reference standard, 0.2 mg/mL, 60 µL) were added to 50 mg of the powdered sample. After stirring for 5 min, the mixture was centrifuged (16,000× *g*, 0, 5 min). Ultrapure water (400 µL) was then added to 800 µL of the supernatant, followed by stirring for 1 min and then centrifugation (16,000× *g*, 0 °C, 5 min). A 400-µL aliquot of the supernatant was concentrated for 1 h using a centrifugal evaporator (CVE-2000, Eyela, Japan), before being freeze-dried overnight. Methoxyamine hydrochloride solubilized with pyridine (20 mg/mL, 50 µL) was added to the freeze-dried sample, and oxime was formed in a reaction at 30 °C for 90 min. Subsequently, 100 µL of MSTFA was added, and trimethylsilylation was conducted by reaction at 37 °C for 30 min. The derivatized samples were then subjected to GC-MS analysis.

#### 4.4.2. Analytical Conditions

The GC-MS device used was a GCMS-QP2010 Ultra System (Shimadzu, Japan), and the GC column was an Agilent J&W DB-5 (length, 30 m; internal diameter, 0.25 mm; film thickness, 1.00 µm; Agilent Technologies, USA). The GC oven temperature was set at 100 °C for 4 min before being increased to 320 °C at 10 °C/min, and held for 11 min at 320 °C. The injection port temperature was 280 °C. A derivatized sample (1 µL) was injected in split-injection mode with a split ratio of 10:1. Helium was the carrier gas, and its linear velocity was kept constant (39.0 cm/s). The purge flow rate was 5 mL/min. Quadrupoles were used for MS mass separation, and electron impact was used for ionization. The ion source temperature was 200 °C, the interface temperature was 280 °C, and the ionization voltage was 70 eV. The measurements were taken in scan mode in the range 45–600 *m*/*z*.

#### 4.4.3. Data Processing

Retention time correction (retention index) was conducted based on the retention time of a standard alkane-series mixture (C-6 to C-33) by applying the “automatic adjustment of retention time” function of Shimadzu GCMSsolution software. Peak annotation was performed using the GC/MS Metabolite Component Database Ver. 2 (Shimadzu Co., Kyoto, Japan), which contains a mass spectral library. Peaks were annotated when they had a similarity index of >80 and a target ion with a confirmation ion ratio of ≥50% in absolute tolerance.

### 4.5. Multivariate Analysis

SIMCA 14 (MKS Instruments, USA) was used for the multivariate analysis. The values of each taste attribute obtained by the electronic tongue and each metabolic component identified by GC-MS analysis were averaged for each sample group (*n* = 3). The metabolites identified by GC-MS analysis were treated as *x* variables, and the taste values obtained by the electronic tongue were treated as *y* variables. Only compounds that were detected in all three samples were included in the dataset. PCA-X or Y, which are unsupervised learning analyses without *y* or *x* variables, respectively, were conducted with UV or Par pretreatment (normalization) to identify the differences in metabolic components or taste-attribute profiles between the samples. Next, O2PLS was used to investigate the relationships between the *x* and *y* variables. O2PLS not only predicts *y* to *x*, as does PLS and OPLS, but can also predict *y* from *x* [21,22], which is ideal for analyzing datasets containing multiple *y* variables. The model obtained by the O2PLS analysis was statistically significant (*R^2^Y* ≥ 0.65 and *Q^2^* ≥ 0.5) [23].

## 5. Conclusions

In this study, we evaluated the taste of yellowtail muscle by metabolic profiling combined with GC-MS analysis and an electronic tongue. We identified differences in taste attributes between types of yellowtail muscle, as well as metabolites that were significantly related to taste attributes. OM storage had no effect on taste, and there were no significant relationships with metabolites. However, in DM, storage affected the taste attribute “irritant,” which was related to a metabolic component. Therefore, this method was very effective in evaluating the taste of fish meat.

## Supplementary Materials

The following are available online, Table S1: List of taste attributes and metabolites, Figure S1: Score plots (A, B) and loading plots (C, D) obtained by a principal components analysis (PCA)-X of the metabolic profiles, Figure S2: Score plots (A, B, E, F) and loading plots (C, D, G, H) obtained by a principal components analysis (PCA)-X of metabolic profiles in dark (A–D) and ordinary (E–H) muscle, Figure S3: Two-way orthogonal projections to latent structures analysis with pareto scaling as a pretreatment, Figure S4: Regression analyses of the predictive model for each *y* variable, Figure S5: Two-way orthogonal projections to latent structures analysis using *y* variables for which significant models were created in Figures S3 and S4, Figure S6: Two-way orthogonal projections to latent structures analysis with unit variance scaling as a pretreatment of dark muscle samples, Figure S7: Two-way orthogonal projections to latent structures analysis with pareto scaling as a pretreatment in dark muscle samples, Figure S8: Regression analyses of the predictive model for each *y* variable in Figure S7, Figure S9: Two-way orthogonal projections to latent structures analysis of ordinary muscle samples.
