# Supplementary material for: Taste Evaluation of Yellowtail (Seriola Quinqueradiata) Ordinary and Dark Muscle by Metabolic Profiling
Source: Molecules. 2019 Jul 15;24(14):2574. doi: 10.3390/molecules24142574 (PMC6680873; doi:10.3390/molecules24142574)
Supplement: Supplementary file 1 [file molecules-24-02574-s001.zip › Supplementary Figures.pdf]

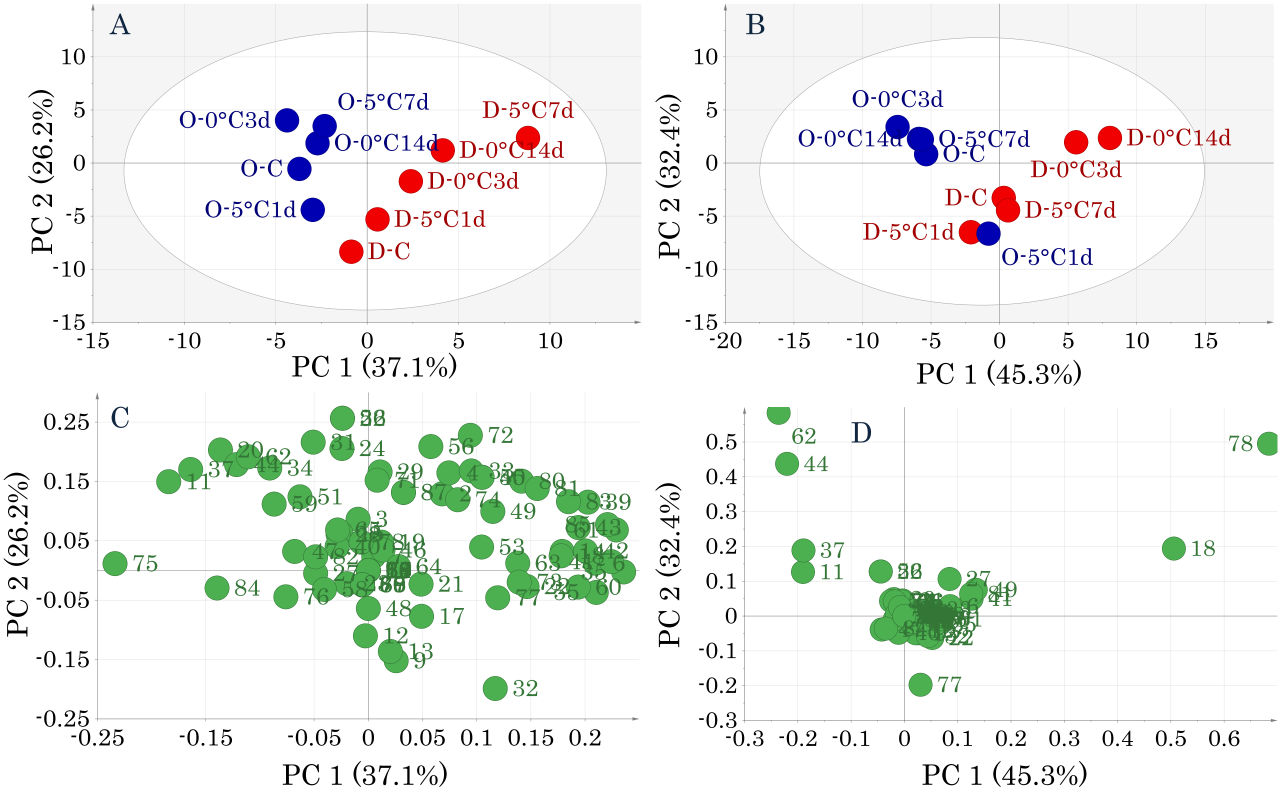

Supplementary Figure S1 Score plots (**A, B**) and loading plots (**C, D**) obtained by a principal components analysis (PCA)-X of the metabolic profiles. Data were pretreated with unit variance scaling (**A, C**) and pareto scaling (**B, D**). Symbols in score plots (**A, B**) indicate sample IDs (Table S1). Numbers in loading plots (**C, D**) indicate *x* variable IDs (Table S1).

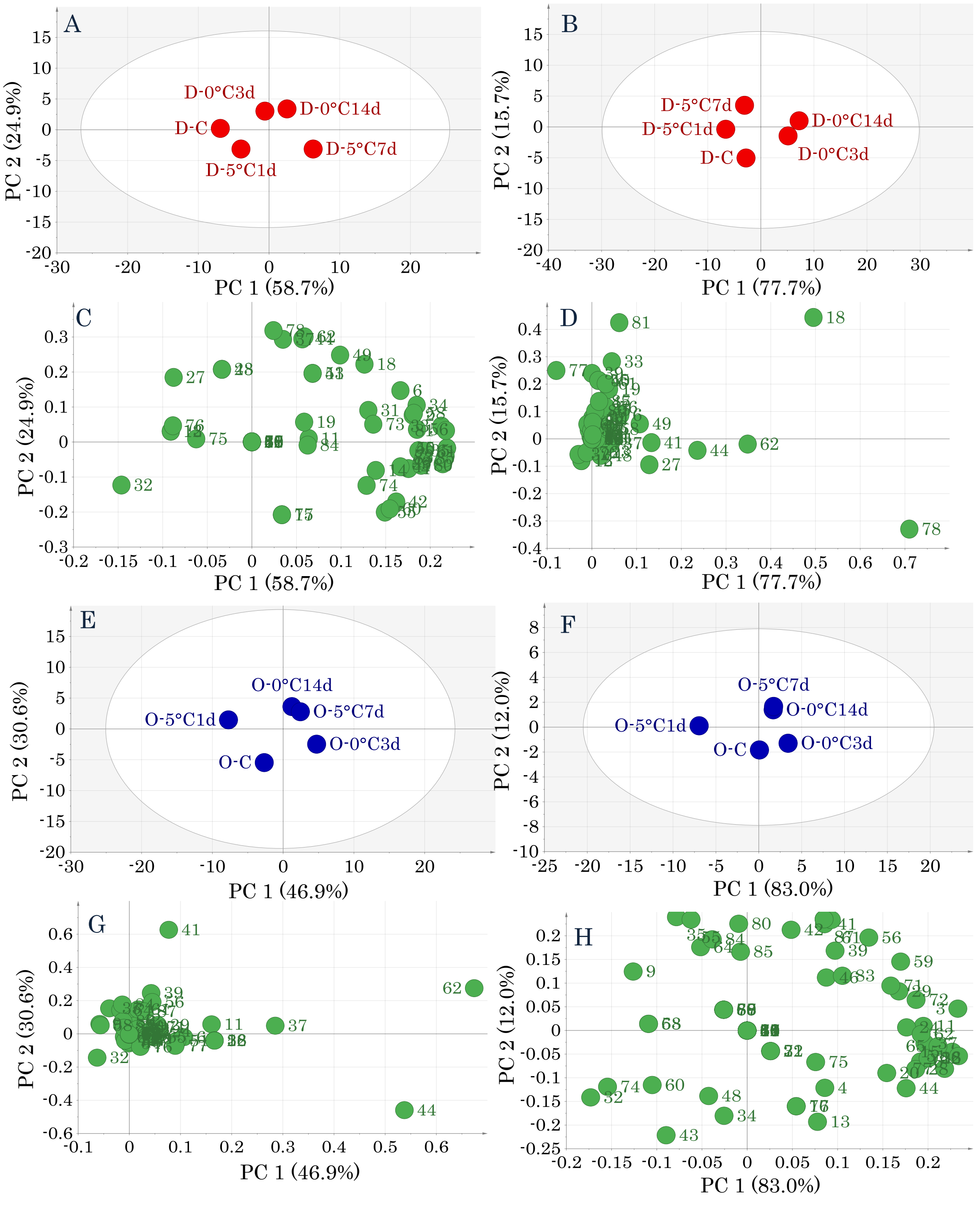

Supplementary Figure S2 Score plots (**A, B, E, F**) and loading plots (**C, D, G, H**) obtained by a principal components analysis (PCA)-X of metabolic profiles in dark (**A-D**) and ordinary (**E-H**) muscle. Data were pretreated with unit variance scaling (**A, C, E, G**) and pareto scaling (**B, D, F, H**). Symbols in score plots (**A, B, E, F**) indicate sample IDs (Table S1). Numbers in loading plots (**C, D, G, H**) indicate *x* variable IDs (Table S1).

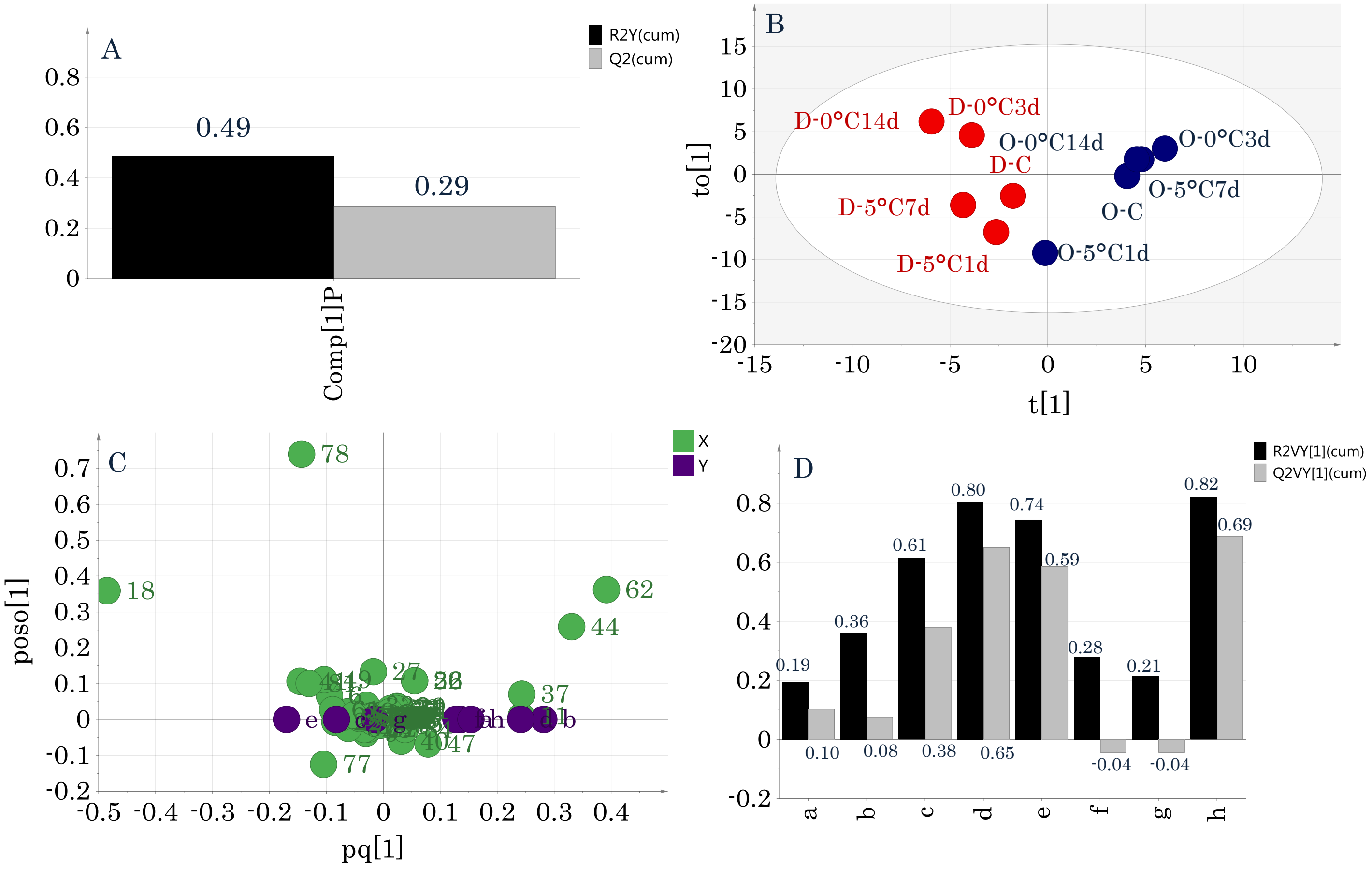

Supplementary Figure S3 Two-way orthogonal projections to latent structures analysis with pareto scaling as a pretreatment. **(A)** Evaluation of the model when Autofit was applied. Model dimensionality was 1+1+3. **(B)** Score plot. Symbols indicate sample IDs (Table S1). **(C)** Loading plot. Numbers and letters indicate variable IDs (Table S1). **(D)** Evaluation of the predictive model for each *y* variable. Letters on the x-axis indicate *y* variable IDs (Table S1).

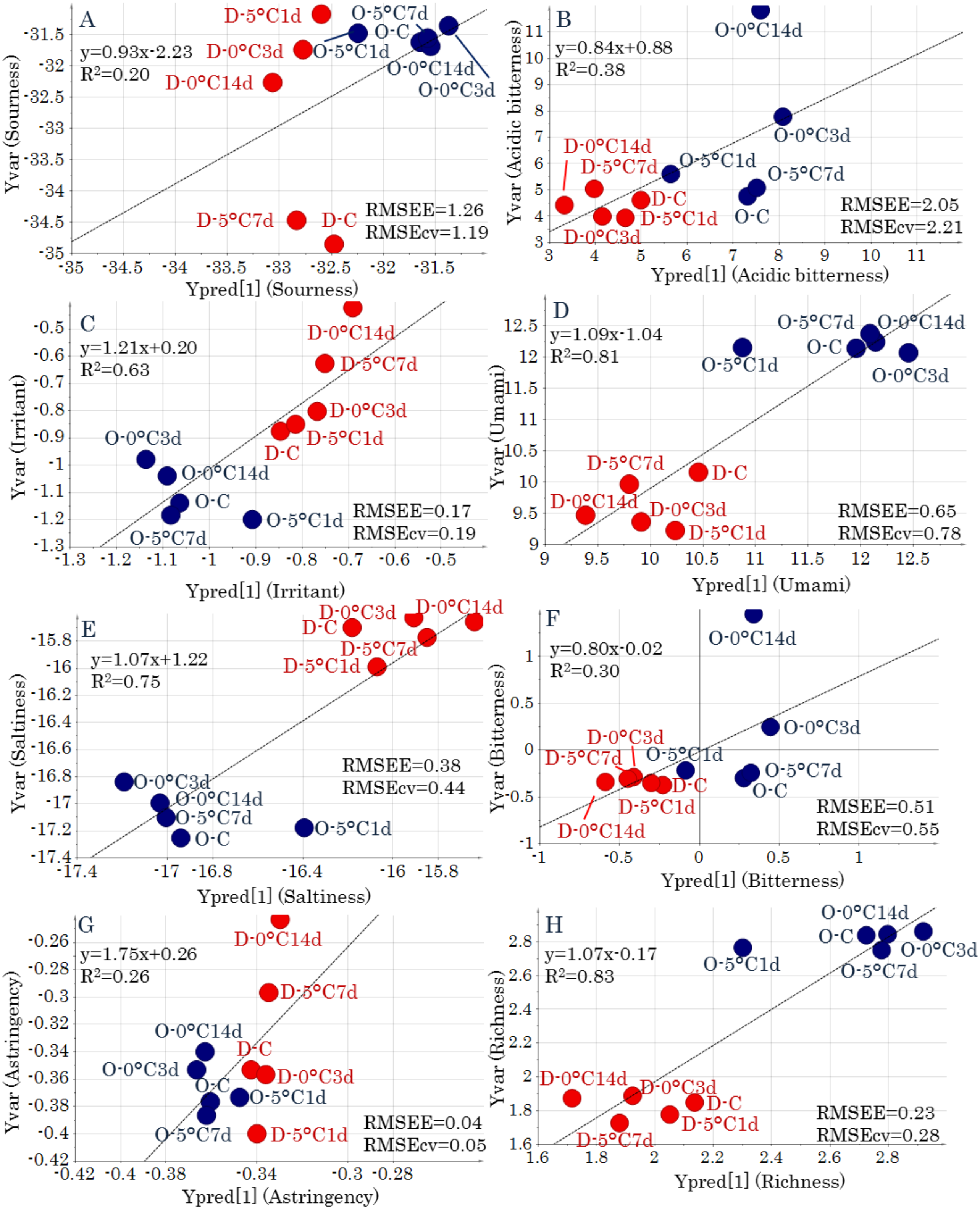

Supplementary Figure S4 Regression analyses of the predictive model for each *y* variable. **(A)** Sourness. **(B)** Acidic bitterness. **(C)** Irritant. **(D)** Umami. **(E)** Saltiness. **(F)** Bitterness. **(G)** Astringency. **(H)** Richness. Symbols indicate sample IDs (Table S1). *RMSEE*, root mean square errors of estimation; *RMSEcv*, root mean square errors of cross-validation.

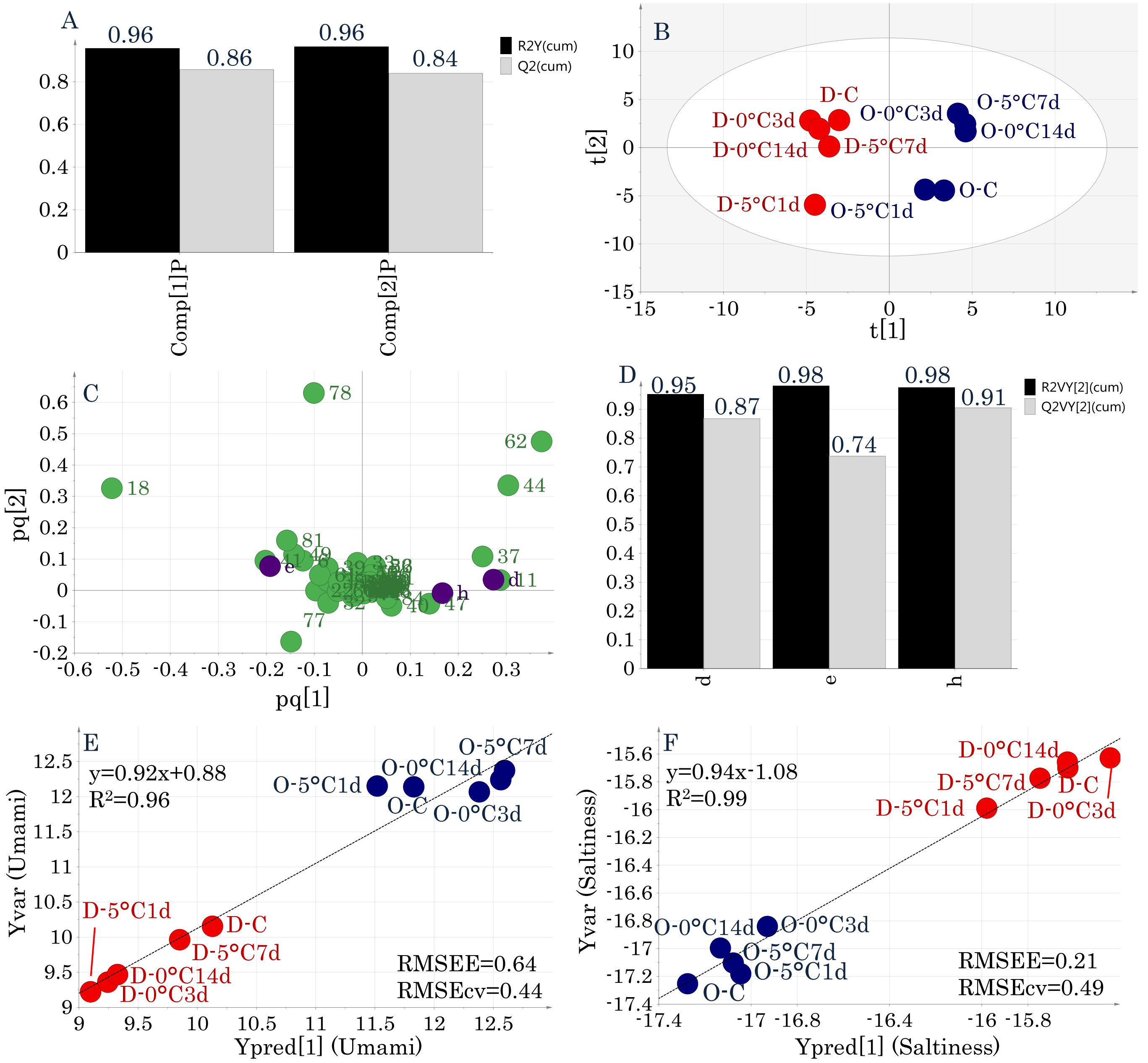

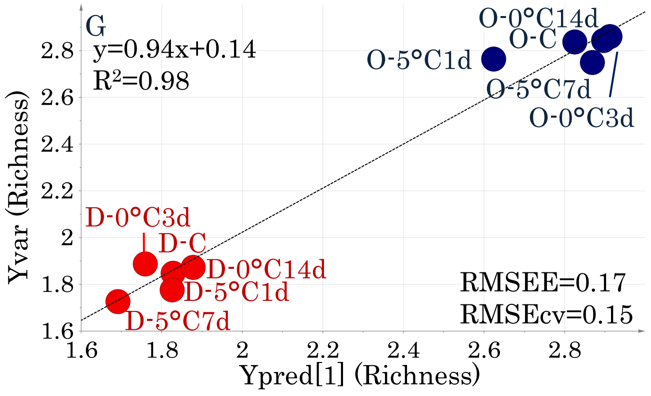

Supplementary Figure S5 Two-way orthogonal projections to latent structures analysis using *y* variables for which significant models were created in Figures 5 and 6. (**A**) Evaluation of the model when Autofit was applied. (**B**) Score plot. Symbols indicate sample IDs (Table S1). (**C**) Loading plot. Numbers and letters indicate variable IDs (Table S1). (**D**) Evaluation of the predictive model for each *y* variable. Letters on the x-axis indicate *y* variable IDs (Table S1). (**E–G**) Regression analyses of the predictive model for each *y* variable. Symbols indicate sample IDs (Table S1). *RMSEE*, root mean square errors of estimation; *RMSEcv*, root mean square errors of cross-validation. (**E**) Umami. (**F**) Saltiness. (**G**) Richness.

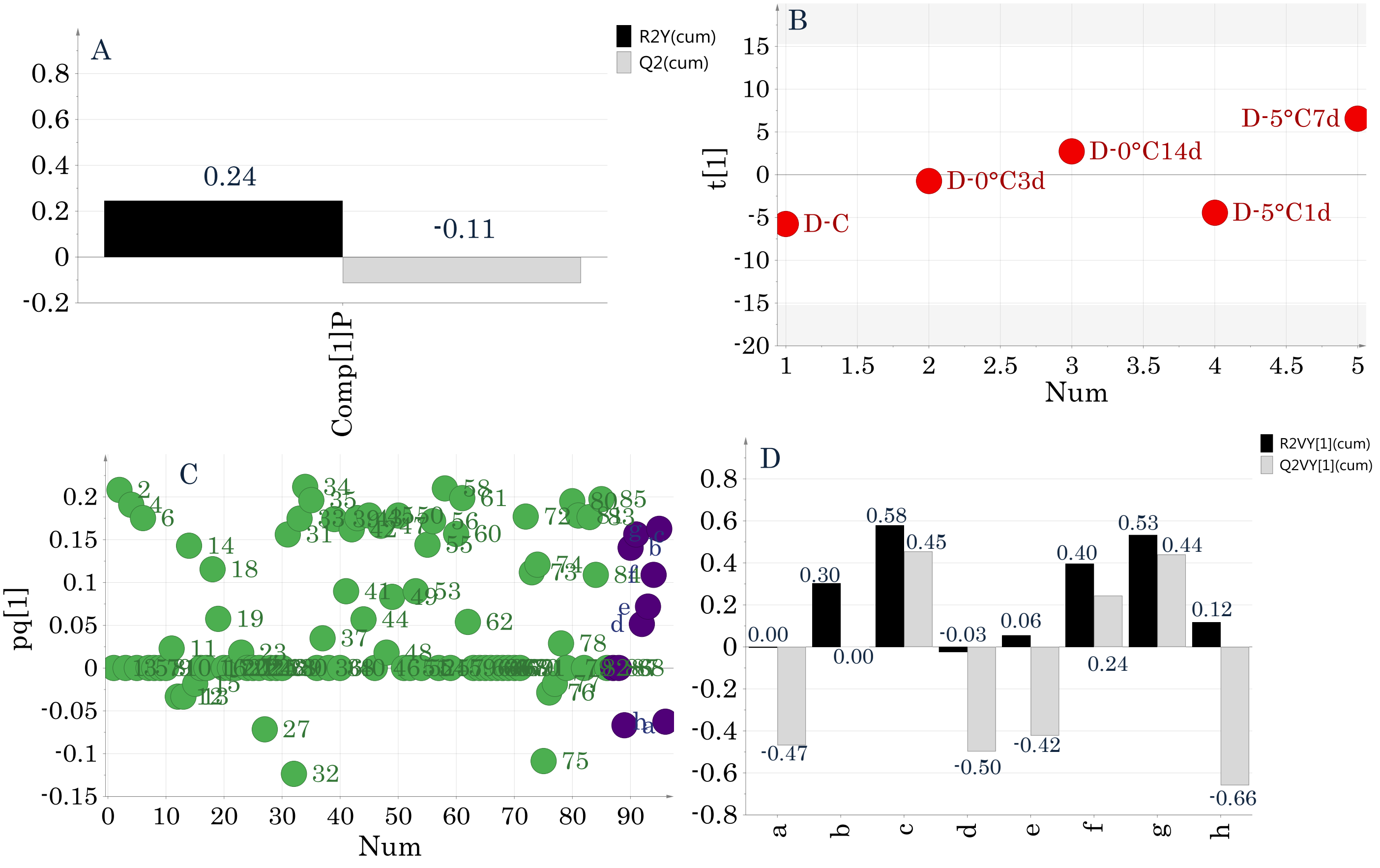

Supplementary Figure S6 Two-way orthogonal projections to latent structures analysis with unit variance scaling as a pretreatment in dark muscle samples. (**A**) Evaluation of the model when Autofit was applied. (**B**) Score plot. Symbols indicate sample IDs (Table S1). (**C**) Loading plot. Numbers and letters indicate variable IDs (Table S1). (**D**) Evaluation of the predictive model for each *y* variable. Letters on the x-axis indicate *y* variable IDs (Table S1).

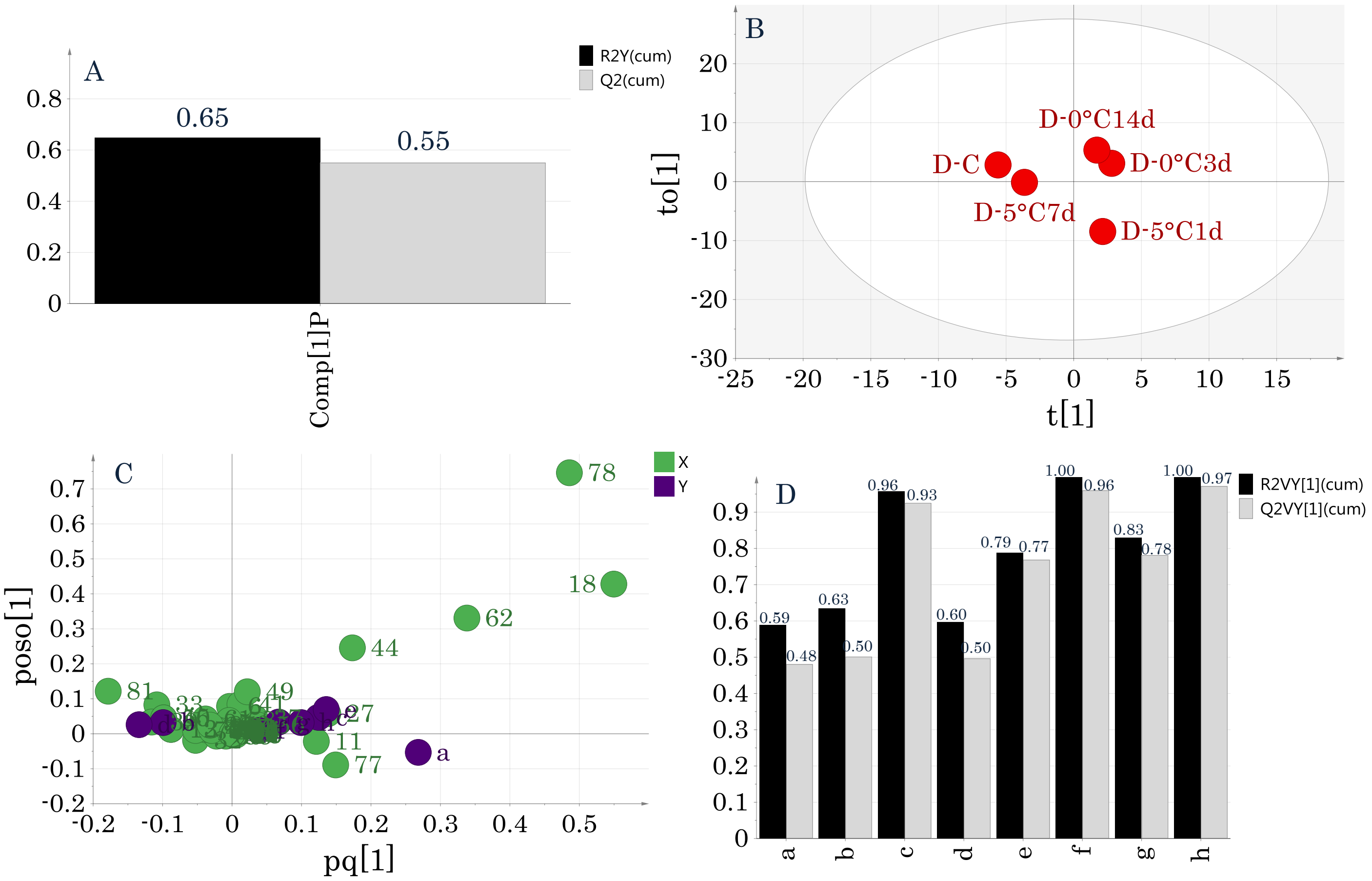

Supplementary Figure S7 Two-way orthogonal projections to latent structures analysis with pareto scaling as a pretreatment in dark muscle samples. **(A)** Evaluation of the model when Autofit was applied. Model dimensionality was 1+3+3. **(B)** Score plot. Symbols indicate sample IDs (Table S1). **(C)** Loading plot. Numbers and letters indicate variable IDs (Table S1). **(D)** Evaluation of the predictive model for each *y* variable. Letters on the x-axis indicate *y* variable IDs (Table S1).

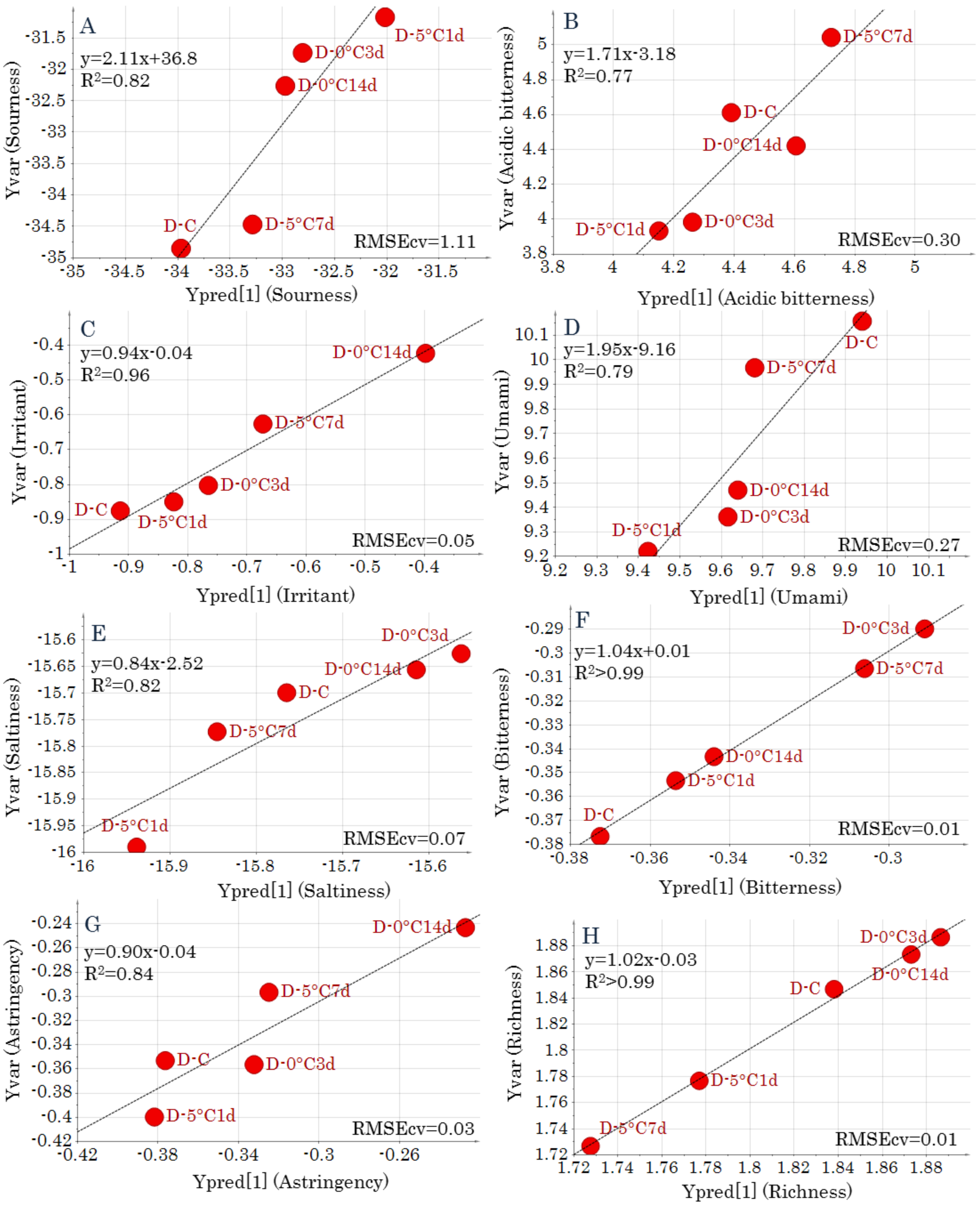

Supplementary Figure S8 Regression analyses of the predictive model for each y variable in Figure S7. **(A)** Sourness. **(B)** Acidic bitterness. **(C)** Irritant. **(D)** Umami. **(E)** Saltiness. **(F)** Bitterness. **(G)** Astringency. **(H)** Richness. Symbols indicate sample IDs (Table S1). *RMSEcv*, root mean square errors of cross-validation.

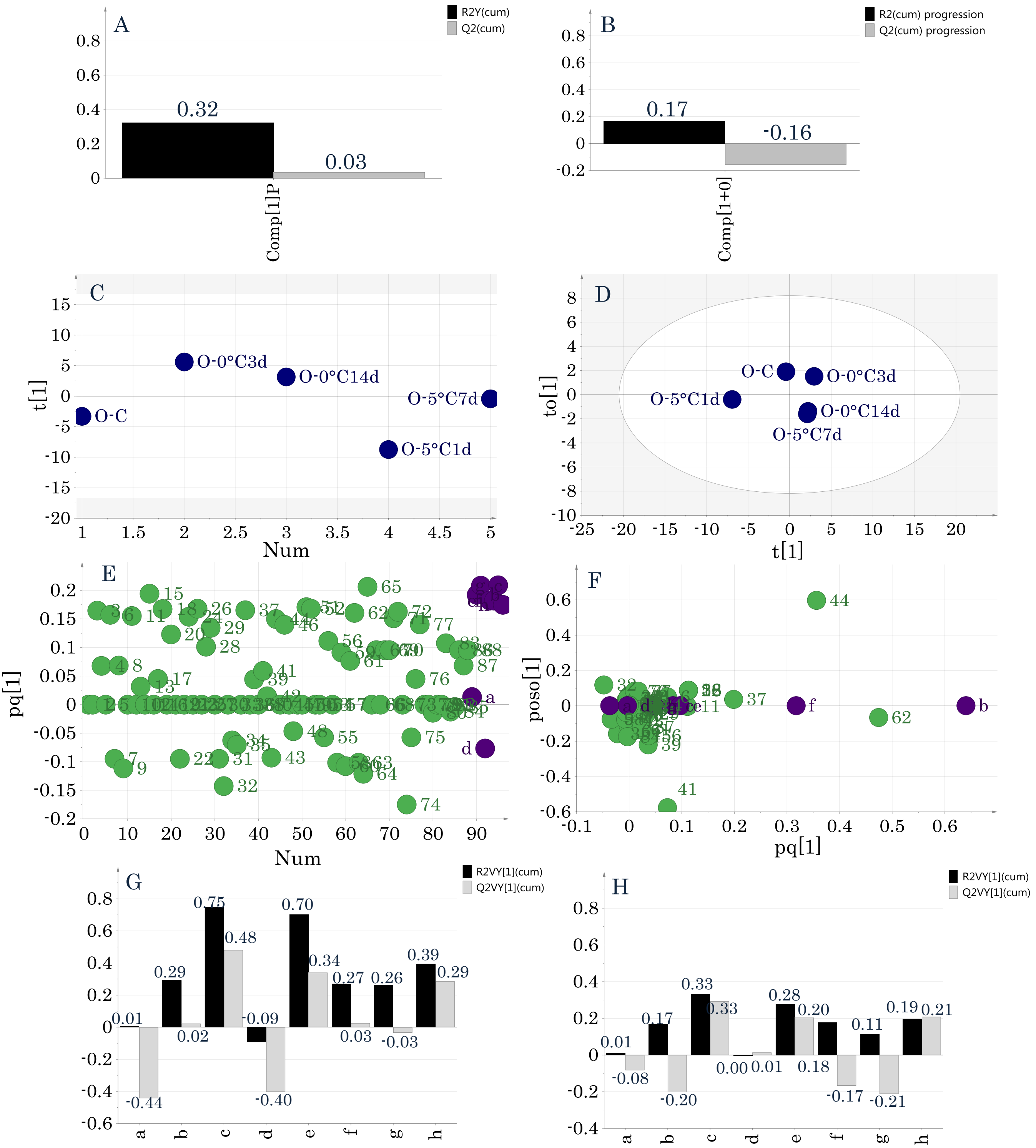

Supplementary Figure S9 Two-way orthogonal projections to latent structures analysis of ordinary muscle samples. Data were pretreated with unit variance scaling (**A, C, E, G**) and pareto scaling (**B, D, F, H**). (**A, B**) Evaluation of the model when Autofit was applied. Model dimensionality was 1+0+1 (A) and 1+1+0 (B). (**C, D**) Score plot. Symbols indicate sample IDs (Table S1). (**E, F**) Loading plot. Numbers and letters indicate variable IDs (Table S1). (**G, H**) Evaluation of the predictive model for each y variable. Letters on the x-axis indicate y variable IDs (Table S1).
